# Anterior Minimally Invasive Approach (AMIS) for Total Hip Arthroplasty: Analysis of the First 1000 Consecutive Patients Operated at a High Volume Center

**DOI:** 10.3390/jcm13092617

**Published:** 2024-04-29

**Authors:** Cesare Faldini, Valentino Rossomando, Matteo Brunello, Claudio D’Agostino, Federico Ruta, Federico Pilla, Francesco Traina, Alberto Di Martino

**Affiliations:** 1 1st Orthopaedic Department, IRCCS—Istituto Ortopedico Rizzoli, via Giulio Cesare Pupilli, 1, 40136 Bologna, Italy; cesare.faldini@ior.it (C.F.); valentino.rossomando@ior.it (V.R.); matteo.brunello@ior.it (M.B.); claudio.dagostino@ior.it (C.D.); federico.ruta@ior.it (F.R.); federico.pilla@ior.it (F.P.); 2 Department of Biomedical and Neuromotor Science-DIBINEM, University of Bologna, 40126 Bologna, Italy; francesco.traina@ior.it; 3 Ortopedia, Traumatologia e Chirurgia Protesica e dei Reimpianti di Anca e Ginocchio, IRCCS Istituto Ortopedico Rizzoli, 40136 Bologna, Italy

**Keywords:** direct anterior approach, total hip arthroplasty, anterior minimally invasive surgical technique, outcomes, survival, femoral-cutaneous nerve apraxia

## Abstract

(1) **Background:** Direct anterior approach (DAA) has recently acquired popularity through improvements such as the anterior minimally invasive surgical technique (AMIS). This retrospective study examines the first 1000 consecutive THAs performed utilizing the AMIS approach in a high-volume center between 2012 and 2017. (2) **Methods:** 1000 consecutive THAs performed at a single institution utilizing the AMIS approach were retrospectively analyzed with a minimum five-year follow-up. Full evaluation of demographic information, clinical parameters, intraoperative complications, and radiological examinations are reported. (3) **Results:** Overall complication rate was 9.4% (94/1000), including 8 dislocations, 57 femoral-cutaneous nerve injuries, 12 intraoperative femoral fractures, 9 infections and 8 leg length discrepancy. Implant survival rates were 98.5% at 1 year, 97.5% at 3 years, 97% at 5 years, and 95.3% at 7 years. Causes of failure included periprosthetic fractures (0.8%), implant dislocations (0.6%), septic loosening (0.5%), aseptic mobilizations (0.2%), and symptomatic limb length discrepancies (0.2%). (4) **Conclusions:** Controversies persist around the direct anterior approach (DAA) for THA, primarily regarding the increased complications rate during the learning curve. However, this study advocates for widespread adoption of the DAA approach. The results demonstrate acceptable complication rates and remarkable functional outcomes, affirming its viability in the broader orthopedic patient population.

## 1. Introduction

Total hip arthroplasty (THA) is perceived as the most effective treatment for end-stage hip osteoarthritis (OA). It can be performed through a number of different approaches, including, direct lateral (LA; Bauer), posterior (PA; Kocher-Langenbeck), posterolateral (PLA; Gibson-Moore), anterolateral (ALA; Rottinger), and direct anterior (DAA; Smith-Petersen; Hueter, Matta) [1,2]; however, the superiority of a surgical approach over the others for THA performance is a controversial issue. DAA for THA has gained popularity over the last few years [3,4,5,6,7,8,9].

Carl Hueter described the use of the interval between the tensor fasciae latae and the sartorious muscle to gain access to the hip joint in his 1817 German manuscript “Der Grundriss der Chirurgie [10]”, to manage war injuries and infectious diseases of the femoral head. Smith-Petersen popularized the approach throughout the English-speaking scientific community after describing its use for the open reduction of congenital dislocation of the hip, in 1917.

The modern anterior minimally invasive surgical approach, or AMIS, (Medacta, Switzerland) was developed by F. Laude in 90′ in Paris [6]. This technique was brought and further modified later on in the United States by Matta [2]. The modern anterior minimally invasive surgical approach could require the use of a traction table and dedicated instrumentation with curved retractors, off-setted handles for acetabular reaming and cup implantation, and curved handles for femoral preparation and stem implantation, designed to minimize the extension of the surgical exposure for THA performance through DAA. The evolution in instrumentation was parallel to the evolution of the design of implants; a short corail-type stem has been proposed [11,12]. More recently, this surgical technique has been described for difficult cases once considered less suitable to it [13,14].

The aim of this retrospective study was to analyze the clinical and radiological outcomes of a series of 1000 consecutive THAs performed by a modified minimally-invasive DAA in a single high volume center between 2012 and 2017, (1) to determine survival at a minimum 5 year follow up, (2) to evaluate radiological and clinical outcomes, and perioperative complications.

## 2. Materials and Methods

### 2.1. Study Design and Patients

We retrospectively studied the first 1000 consecutive patients (426 men and 574 women) affected by degenerative arthritis of the hip, either primary or secondary, undergoing THA by AMIS approach between 2012 and 2017, and followed up for a minimum of 5 years. The study was approved by the ethical committee of the authors’ institution (ANT-HIP; CE AVEC:347/2021/Oss/IOR). The surgical technique was performed in supine position using a traction table for all the patients. These were operated on by expert hip surgeons. Inclusion criteria for study enrolment were: (1) age above 18 years, (2) primary and secondary degenerative arthritis, (3) DAA approach. The exclusion criteria were: (1) neurological disorders, (2) revision surgery, (3) simultaneous bilateral hip replacement, (4) THA implanted following tumor resections.

### 2.2. Surgical Technique

**Figure 1:**
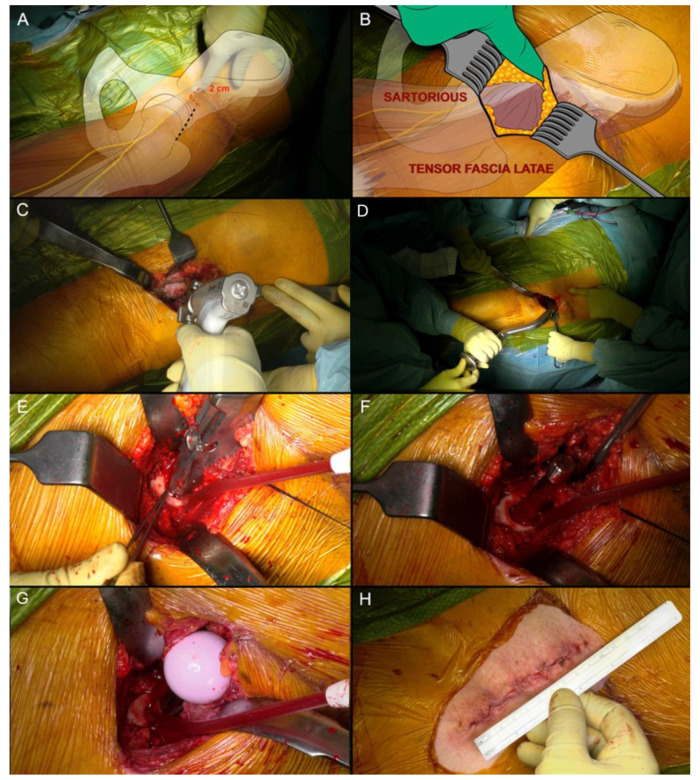
(**A**) Surgical incision; (**B**) Intermuscular plane dissection; (**C**) Femoral neck ostetotomy; (**D**) Cup impaction; (**E**) Femoral broaching; (**F**) Stem insertion; (**G**) Head Insertion; (**H**) Surgical wound.

**Figure 2:**
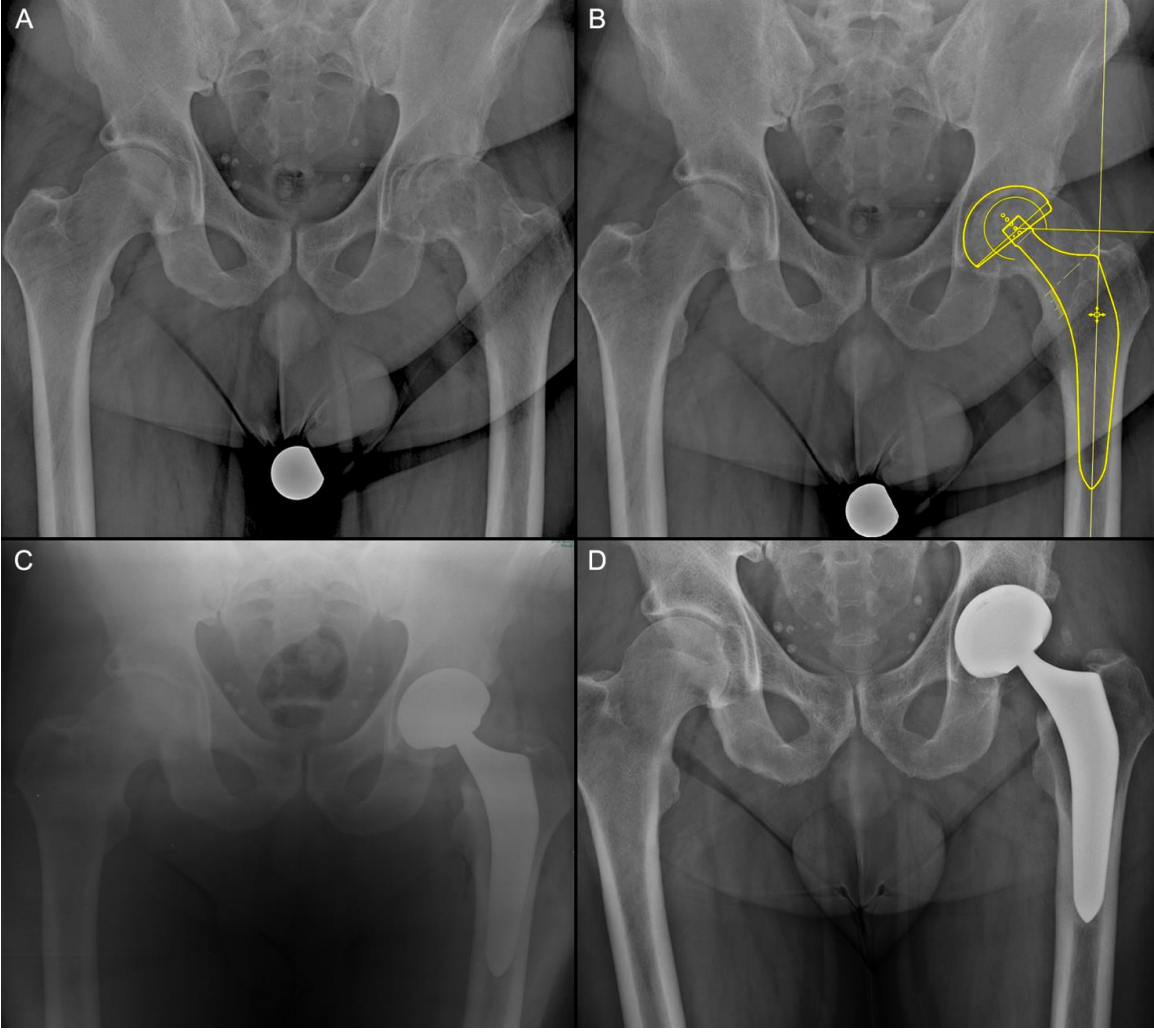
Male, 67y.o. (**A**) Preoperative Radiograph of the hip and pelvis. (**B**) Digital Templating; (**C**) Post-Operative Radiographic control. (**D**) 7 years follow-up radiograph.

All the patients underwent standing antero-posterior X-rays of the pelvis and frog leg X-rays of the affected hip. Digital pre-operative planning [15] was performed by OrthoView (Materialise, Leuven, Belgium). At surgery, patient was positioned supine with the foot of the operated leg restrained by a boot on a specialized traction table supervised by an un-scrubbed assistant, allowing control of traction, flexion, rotation, adduction and abduction. Before surgery began, the lower limb was positioned with 10° of internal rotation and a slight abduction, and it was kept with approximately 15° of hip flexion by the use of a step. Surgical incision was started 2 cm distally and 2 cm laterally from the anterior superior iliac spine, and it was extended approximately 7–8 cm distally (Figure 1A). Subcutaneous plane dissection was then performed until the fascia of tensor fascia latae muscle (Figure 1B), which was incised longitudinally and slightly lateral over the belly of the tensor fascia latae muscle to avoid damage at the lateral femoral cutaneous nerve. The tensor fascia latae was retracted laterally and the sartorius muscle medially. Branches of the lateral circumflex artery were isolated and ligated in every case. The capsula was totally exposed and gently opened with a seven-shape incision, resulting in a thick proximally reflected flap suspended by a stich. The femoral neck was osteotomized, according to the preoperative planning, 5 mm proximal to the trochanteric tubercle (Figure 1C). The leg was then placed under modest traction and external rotation to enlarge the osteotomy area before the head was extracted using a corkscrew. Acetabular preparation was then performed using a chamfered reamer, to limit impingement on soft tissues. Two screws were used if required (Figure 1D). To prepare the femur (Figure 1E–G) the leg was progressively external rotated, extended and adducted. Simultaneously, a release of pubofemoral and ileofemural ligaments, and of the posterior-medial capsule was performed to promote the elevation and external rotation of the femur. The capsule was sutured, and an intra-articular drainage was used in every case. A final X-ray check was performed at the end of the surgery (Figure 2).

**Figure 3:**
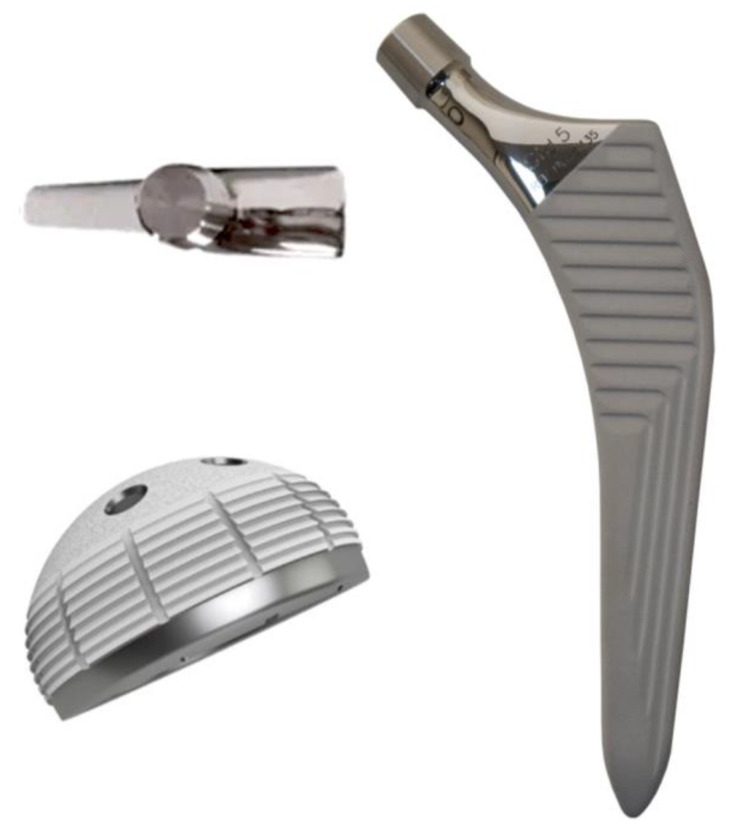
The corail-like stems used, AMIS stem and the elliptic cup, Versafit CC Trio (Medacta^®^).

The same implant design and type of instruments were used for all the surgeries: Versafit CC Trio (Medacta, Castel San Pietro, Switzerland) for the cup, a hydroxipatite-coated elliptical cup, and AMIS stem-H or AMIS stem-P (Medacta, Castel San Pietro, Switzerland) for the stem, a straight, triple tapered, hydroxyapatite-coated not cemented stem (Figure 3). The P variant of the stem represents the second generation of the H stem, with modified metaphyseal coating, to promote primary stability. Joint coupling components were Biolox Delta (CeramTec, Plochingen Germany) ceramic-on-ceramic in 95.7% of hips, and ceramic-on-XLPE in 4.3% of patients.

### 2.3. Postoperative Care

To facilitate a faster recovery after surgery, acetaminophen and tramadol were supplied every 8 h in the first postoperative day and then if required by the patient. The drain was removed 24 h following surgery. Patients were hospitalized after surgery to begin the rehabilitation protocol which began on the same day, or the day after the surgery, if the surgery was performed in the afternoon: patients were sit after resolution of the epidural anesthesia, and they were helped to stand up as soon as possible. Isometric exercises were started already in bed. Stair climbing was allowed through crutches with progressive weight-bearing, and it was set as a goal on the second to third day after THA. The patient was discharged or transferred to a rehabilitation facility if able to walk with crutches, climb stairs, dress themselves, and use the restroom. Thromboprophylaxis by subcutaneous low molecular weight heparin was administered 12 h after index surgery, and then daily for at least five weeks after.

### 2.4. Clinical and Radiological Evaluation

Patients were retrospectively studied by collecting demographic and clinical parameters, and data were taken from the medical records: age at surgery, BMI, diagnosis at surgery, American Society of Anesthesiologists (ASA) score, incision length, operative time, intraoperative blood loss, number of patients requiring transfusion, in-hospital stay, and time to stair climbing. For each patient, intra-operative and peri-operative complications were recorded and pooled. Early complications within one month after surgery were also recorded. Harris hip score (HHS) was calculated both preoperatively and in the follow-up period using the Italian validated version [16]. At follow-up visits, performed at 1 month, 3 month, 6 month, 1 year and then yearly, postoperative radiographs including anteroposterior and axial view of the hip were performed. Radiolucency and periprosthetic osteolysis were identified using Gruen femoral zones for the stem and the DeLee and Charnley’s acetabular zones [17]. Criteria for osteolysis were defined as the radiographic appearance of a focused area of bone loss greater than 2 mm wide. If serial radiographs revealed subsidence greater than 3 mm compared to the immediate postoperative radiograph, varus or valgus tilting, the femoral stem was termed loose. Stem alignment was considered good when the angle between the axis of the stem and that of the femur was 0°± 5°; above or below the range, the implant alignment was considered in varus or valgus, respectively. Cup inclination was considered normal when 40° ± 10° [18]. The radiographic analysis was independently performed by three surgeons (one senior and two residents), who were not involved in the surgery.

### 2.5. Statistical Analysis

Kaplan-Meier and SPSS 18.0 statistical software (SPSS, Chicago, IL, USA) were used to determine implant survival with 95% confidence interval (CI). Revision surgery for any reason was specified as the end point. The time range between the date of the first implantation and the date of the revision procedure was used to calculate the time to revision. The preoperative average HHS and those at the most recent follow-up were compared using a paired *t*-test. Significance was defined as *p* < 0.05. Study variables were examined and compared between groups. Continuous data points were expressed in terms of its average and standard deviation.

## 3. Results

### 3.1. Demographics

From a total of 1312 primary THAs performed from 1 January 2012 to 31 December 2017, the first 1000 consecutive implants in 1000 patients (426 males and 574 females; 42.6% M and 57.4% F) that matched the inclusion criteria were enrolled. Average age was 74 years old; 6 patients were under 40 years old, 41 patients were between 40–49 years old, 139 were between 50–59 years old, 302 were between 60–69 years old, 404 were between 70–79 years old, and 108 patients were over 80 years old (Table 1).

Results of BMI calculation were available in 76% of patients; BMI showed an average value of 26.4 ± 3.7 kg/m^2^. 35.7% (*n* = 357/1000) of patients were overweight, followed by normal weight (26.5%) (*n* = 265/1000), obese (12.5%) (*n* = 125/1000), and underweight (1.3%) (*n* = 13/1000). 62% (621/1000) of patients were ASA score II, 23% (229) were ASA I and 15% (150) were ASA III. Indication for surgery, as per inclusion criteria, were primary degenerative osteoarthritis in 72.5% of patients, followed by secondary osteoarthritis after femoral neck fracture in 11.3% and osteonecrosis of the femoral head in 7.1% (Table 2).

Regarding surgical-related data, average operative time was 79 ± 20 min. Surgical incision averaged 7.6 ± 2.3 cm. Intraoperative blood loss was 210.3 ± 191 mL; as regards transfusions of Blood Units, 78.6% of patients did not require transfusion, 14.5% had one unit of blood, 6.0% required 2 units, and 0.9% more than two transfusions (Table 3).

### 3.2. Intra-Operative and Early Complications

An overall rate of intra-operative and early complications of 9.4% (94/1000) was observed. Specifically, 8 dislocations were recorded, 2 treated by close reduction, while 6 required open reduction. 57 femoral-cutaneous nerve injury were recorded, 46 of which recovered between 6 and 12 months from surgery. 12 intraoperative femoral fractures occurred, all managed during the same surgery by metal wiring. 6 superficial wound infections and 3 deep infections occurred within 30 days after surgery. 3 superficial wound infections resolved within 7 days with administration of antibiotics, the others needed a superficial surgical debridement followed by antibiotic therapy; all the 3 early deep infections, which presented an evident subfascial fluid collection and/or intra-articular fistula, underwent debridement, antibiotic therapy and implant retention (DAIR), with a good outcome within 1 month after revision surgery. 8 radiographic and/or clinical limb length discrepancies (LLD) above 10 mm were recorded. Specifically, 7 LLDs in plus and 1 LLD in minus were confirmed. Of these eight patients, 2 were reoperated with revision of the head length (Table 4).

### 3.3. Implants Survival and Failures

**Figure 4:**
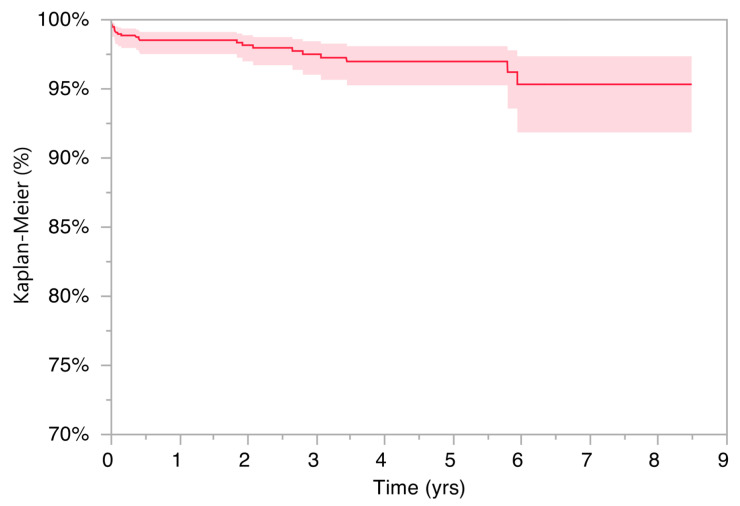
Survival rate of the first 1000 DAA implants; end-point revision surgery.

The average follow-up was 8 years. The different survivals at 1, 3, 5 and 7 years after surgery were then analyzed. 1 year after surgery survival was 98.5% (range 97.5–99.1), at 3 years it was 97.5% (range 96.0–98.4), at 5 years 97% (range 95.3–98.1) at 7 years 95.3% (range 91.8–97.4) (Figure 4).

A total of 23 failures occurred, requiring revision surgery. Specifically, 8 periprosthetic fractures (0.8%), 5 septic loosening (0.5%), 6 implant dislocations (0.6%), 3 aseptic mobilizations (0.3%) and 1 global aseptic mobilization (0.1%), 2 symptomatic limb length discrepancies (LLD) (Table 5).

The analysis of failures (Table 6) showed that 10 out of 23 patients (43.5%) underwent stem and head revision, and two patients (8.7%) had cup, liner and head revision. Two patients (8.7%) underwent head revision alone, three (13%) had liner and head revision. Two patients (8.7%) were explanted of both the femoral and acetabular components for deep infection. One patient (2.3%) underwent only stem revision, one had (2.3%) liner, head and stem revision, one (2.3%) had cup and liner revision, and only one (2.3%) patient was subjected to complete revision of all the implant components.

### 3.4. Clinical and Radiographic Outcomes

The average HHS significantly improved from 52.1 points (range 26–79) preoperatively, to 86.4 points (range 55–96) at the last available follow-up (*p* < 0.001).

Radiologically, cup inclination showed an average placement of 33.1°+/−9°, being within the safe zone in 739 hips (73.9%); 261 (26.1%) implants had acetabular cup inclination outside the safe zone, being below it in 225, and above it in 36 implants.

In 12 and 20 hips, respectively, radiolucent lines were seen around the cup and stem. 15 hips (1.5%) showed stem subsidence, and 10 of those had stem loosening and required femoral revision. Stem subsidence was not progressive in the remaining 5 hips. The median value for stem alignment was 0° with a slight-varus position displayed in 70 patients and a slight-valgus position in 20 patients, within the tolerance range in all patients. In 5 patients, a varus stem was observed and in 2 cases it was valgus outside the tolerance range.

## 4. Discussion

The most important findings of the study were a 7-year survival of 95.3% and a rate of revision surgery during follow up of 2.3%. The overall complication rate both intra- and postoperatively was 9.4% with neuroapraxia of the LFC nerve and implant dislocation being the two most common.

Several limits of the current study should be acknowledged. First and most important, being the current study a report of the first 1000 implants, surgeons were at the beginning of their practice and learning curve; therefore, a higher rate of complications could be observed; however, this is consistent with the real practice of orthopedic surgeons. Moreover, the study cohort was extremely heterogeneous, still representing a good sample of the surgical population commonly referring to an orthopedic surgeon.

DAA for THA has gained popularity among hip surgeons in recent years; however, surgeons still fear the learning curve [19], which was estimated to be between 50 and 100 surgeries [20].

In order to assess the outcomes of DAA in a high-volume orthopedic center in terms of survival, causes of failures, intraoperative complications, and radiographic outcomes, the current study reports on the first 1000 consecutive THAs carried out using uniform surgical technique and implants. When the study’s enrollment concluded in 2017, DAA was used to treat primary and secondary hip osteoarthritis, as well as femoral neck fractures, in the great majority of cases (98% of all primary THA implants). Prior to 2012, every senior surgeon in the department had received extensive education in posterolateral or direct lateral approaches to the hip. Starting in 2012, their whole DAA learning curve was covered by the current study.

Abundance of scientific literature about the risks and benefits of DAA pushed towards the implementation of DAA at the Authors’ institution. Indeed, many studies showed that early postoperative functional recovery was faster [21] when compared to other approaches [22,23], recognizing that after a few months the differences in functional recovery disappear [24,25]. In fact, in the prospective randomized clinical study by Barrett et al. [24], THAs performed by DAA and PL approach were compared, and they showed how post-operative recovery was faster with DAA in the first 6 weeks after surgery, although differences completely disappeared at 6 months. Moreover, a lower incidence of bleeding, reduced physical therapy and hospitalization time were demonstrated when DAA was compared to PL and DL approaches [26,27,28,29].

We assessed implant survival, reasons for failure, and radiographic and clinical outcomes with a minimum of five years of follow-up, concentrating our investigation on both early and long-term outcomes. Lower than earlier findings, the overall failure rate in the current study that necessitated reoperation was reported to be 2.3%. An analysis of the DAA results was subsequently conducted on a comparable cohort of 1329 hips. Aggarwal et al. [30] conducted a retrospective cohort study comparing the five techniques utilized for THA at their institution (PL, DAA, DL, AL, and Northern). Comparable but higher than our findings (2.3%), they discovered that DAA was linked to a failure rate (requiring reoperation) in 8.5%.

The most frequent complications that were encountered included injury to the femoral-cutaneous nerve, dislocations and intraoperative femoral fractures. Injury to LFCN is relatively frequent, because it typically crosses the incision, and can be severed [31]. According to the modified DAA technique by Faldini et al. [13], the incision of the fasciae is performed over the TFL muscle belly, preventing an accidental lesion of LCFN which in most patients lies alongside the sartorius muscle. This can be responsible for the relatively low complication rate in our patients’ population (0.5%). A similar procedure was described by Den Hartog in 2015, that reported a 0.8% rate of LFCN lesion, similar to our findings [32]. Regarding intraoperative femoral fractures, in the systematic review of Lee et al. [33], an intraoperative fracture rate of approximately 2% was observed on 11,000 THAs performed by DAA, higher than our reported incidence of 1.2%.

As regards dislocation, only 0.6% required reoperation, a data which is comparable with previous findings in literature, averaging 0.5% [2,34].

There are some drawbacks in the surgical technique, such as femoral exposure and difficulty in performing straight reaming [35]. Moreover, when using a straight tapered stem, DAA may be associated with a high incidence of varus stem alignment, mainly during the learning curve [36]. This may also be determined by an insufficient capsule release, which makes femur exposure more challenging. In the current study, only 5 patients (0.5%) presented a varus stem, and 2 had valgus alignment (0.2%), all outside the tolerance range. Such a low incidence of stem malpositioning, justifies the use of dedicated instrumentation with offset handles, in addiction mid-size and dedicated stem allow an easier placement and promote implant positioning and alignment [37].

In DAA, either the standard bed or the traction bed can be used for surgery [38]. The outcomes and complications of the standard table and the traction table DAA are similar [39]. In a standard table, it is possible to perform a manual check of leg length discrepancy (LLD), but an additional scrubbed assistant is required; when using a traction table instead, it is possible to indirectly check LLD by intraoperative fluoroscopy, and an unscrubbed assistant operates the traction: we routinely used this latter option. Sarraj et al. [38] published the only systemic review comparing DAA THA with and without an orthopedic traction table. They did not observe side effects from the use of a traction table, including pudendal nerve palsy; moreover, limb length discrepancy was not analyzed in their review. In our study, overall, 8 patients had LLDs above 1 cm, with only 2 patients (0.2%) requiring reoperation probably because of the meticulous preoperative workout and accurate templating [15].

Some patients might be less suitable for DAA in unexperienced hands, and these include adults with outcomes of developmental dysplasia of the hip, Legg-Calvè-Perthes disease and Slipped capital femoral epiphysis. As outlined by Laude in 2020, THA for DAA on patients with Crowe grade III and IV dysplasia is demanding, but in experienced hands it is associated with satisfactory clinical and radiographic results [14,40]. DAA can also be used efficiently in obese patients due to the reduced anterior width of the body fat at the tight [41]. As highlighted in a previous study, THA performed by DAA is safe in obese patients and shows a low rate of complications and satisfying clinical outcomes [13].

## 5. Conclusions

Due to ongoing debate about DAA’s benefits and drawbacks, many surgeons are reticent to use it in THA procedures for fear of higher complication rates throughout the learning curve. The analysis of a thousand consecutive patients might be representative of the general patient group visiting an orthopedic facility for treatment. According to our research, this technique produced extremely good functional outcomes and an acceptable complication rate.

## Figures and Tables

**Table 1:** Distribution by age.

Age at Surgery (Years)	Patients (*n*)
<40	6
40–49	41
50–59	139
60–69	302
70–79	404
>80	108
Total	1000

**Table 2:** Causes of intervention.

Diagnosis	*n*	%
Primary osteoarthritis	725	72.5%
Secondary osteoarthritis due to femoral neck fracture	113	11.3%
Osteonecrosis of the femoral head	71	7.1%
Developmental dysplasia of the hip	35	3.5%
Post-traumatic arthritis	27	2.7%
Not known	14	1.4%
Rheumatoid arthritis	12	1.2%
Slipped capital femoral epiphysis	3	0.3%

**Table 3:** Surgical data.

Operative Data	
Incision length (cm) *	7.6 ± 2.3
Operative time (min) *	79 ± 20
Intraoperative blood loss (mL) *	210.3 ± 190.6
Blood replacement (U) *	0.7 ± 0.5
N. (%) of patients requiring	
Transfusion	
0	786 (78.6%)
1	145 (14.5%)
2	60 (6%)
>2	9 (0.9%)
In-hospital stay (days) *	7 ± 3
ASA	
I	621 (62%)
II	229 (23%)
III	150 (15%)

* average ± SD.

**Table 4:** A review of the complications occurred in 1000 patients.

Complications	Total
Femoral cutaneous nerve apraxia	57/1000 (5.7%)
Intraoperative femur fracture	12/1000 (1.2%)
Dislocation	8/1000 (0.8%)
Superficial wound infection	6/1000 (0.6%)
Early deep infection	3/1000 (0.3%)
Leg Length >10 mm	8/1000 (0.8%)
Total	94/1000 (9.4%)

**Table 5:** Failures.

Cause of Failure	*n*	Incidence Rate (%)	Cause of Failure (%)
Periprosthetic fracture	8	0.8%	34.8%
Prosthetic dislocation	6	0.6%	26.0%
Septic loosening	5	0.5%	21.7%
Aseptic mobilization	2	0.2%	8.7%
Symptomatic LLD	2	0.2%	8.7%
Total	23	2.3%	100.0%

**Table 6:** Characteristics of revision surgery.

Failures (%)	Cup Revision	Liner Revision	Head Revision	Stem Revision
10 (43.5%)	−	−	+	+
2 (8.7%)	+	+	+	−
2 (8.7%)	−	−	+	−
3 (13%)	−	+	+	−
2 (8.7%)	explanted	explanted	explanted	explanted
1 (4.3%)	−	−	−	+
1 (4.3%)	−	+	+	+
1 (4.3%)	+	+	−	−
1 (4.3%)	+	+	+	+
Tot 23 (100%)				

## Data Availability

All collected data are reported in the current manuscript.

## References

[1] Stolarczyk A., Stolarczyk M., Stępiński P., Dorocińska M.K., Świercz M., Szymczak J., Żarnovsky K., Żuchniewicz A., Maciąg B.M. (2021). The Direct Anterior Approach to Primary Total Hip Replacement: Radiological Analysis in Comparison to Other Approaches. J. Clin. Med..

[2] Matta J.M., Shahrdar C., Ferguson T. (2005). Single-Incision Anterior Approach for Total Hip Arthroplasty on an Orthopaedic Table. Clin. Orthop. Relat. Res..

[3] Rachbauer F., Kain M.S.H., Leunig M. (2009). The History of the Anterior Approach to the Hip. Orthop. Clin. N. Am..

[4] Post Z.D., Orozco F., Diaz-Ledezma C., Hozack W.J., Ong A. (2014). Direct Anterior Approach for Total Hip Arthroplasty. J. Am. Acad. Orthop. Surg..

[5] Lepri A.C., Villano M., Matassi F., Carulli C., Innocenti M., Civinini R. (2020). “Anterolateral” Approach to the Hip: A Systematic Review of the Correct Definition of Terms. Hip Int..

[6] Lesur E., Laude F. (2004). Arthroplastie Totale de Hanche Par Voie Antérieure et Son Évolution Mini-Invasive. EMC—Rhumatol.-Orthopédie.

[7] Regis D., Lugani G., Valentini A., Sandri A., Ambrosini C., Bagnis F., Dorigotti A., Negri S., Magnan B. (2023). Mid-Term Clinical and Radiographic Outcome of Metal-on-Metal Hip Resurfacing through an Anterolateral Approach. Musculoskelet. Surg..

[8] Marchetti P.G. (1974). L Artoplastica Totale d’anca Con La Protesi Monoblocco.

[9] Moldovan F., Moldovan L., Bataga T. (2023). A Comprehensive Research on the Prevalence and Evolution Trend of Orthopedic Surgeries in Romania. Healthcare.

[10] Hueter C. (1880). Grundriss Der Chirurgie.

[11] Noble P.C., Alexander J.W., Lindahl L.J., Yew D.T., Granberry W.M., Tullos H.S. (1988). The Anatomic Basis of Femoral Component Design. Clin. Orthop. Relat. Res..

[12] Rilby K., Nauclér E., Mohaddes M., Kärrholm J. (2022). No Difference in Outcome or Migration but Greater Loss of Bone Mineral Density with the Collum Femoris Preserving Stem Compared with the Corail Stem: A Randomized Controlled Trial with Five-Year Follow-Up. Bone Jt. J..

[13] Di Martino A., Stefanini N., Brunello M., Bordini B., Pilla F., Geraci G., D’Agostino C., Ruta F., Faldini C. (2023). Is the Direct Anterior Approach for Total Hip Arthroplasty Effective in Obese Patients? Early Clinical and Radiographic Results from a Retrospective Comparative Study. Medicina.

[14] Faldini C., Tassinari L., Pederiva D., Rossomando V., Brunello M., Pilla F., Geraci G., Traina F., Di Martino A. (2024). Direct Anterior Approach in Total Hip Arthroplasty for Severe Crowe IV Dysplasia: Retrospective Clinical and Radiological Study. Medicina.

[15] Di Martino A., Rossomando V., Brunello M., D’Agostino C., Pederiva D., Frugiuele J., Pilla F., Faldini C. (2023). How to Perform Correct Templating in Total Hip Replacement. Musculoskelet. Surg..

[16] Banaszkiewicz P.A. (2014). Traumatic Arthritis of the Hip after Dislocation and Acetabular Fractures: Treatment by Mold Arthroplasty: An End-Result Study Using a New Method of Result Evaluation. Classic Papers in Orthopaedics.

[17] DeLee J.G., Charnley J. (1976). Radiological Demarcation of Cemented Sockets in Total Hip Replacement. Clin. Orthop. Relat. Res..

[18] Lewinnek G.E., Lewis J.L., Tarr R., Compere C.L., Zimmerman J.R. (1978). Dislocations after Total Hip-Replacement Arthroplasties. J. Bone Jt. Surg. Am..

[19] Foissey C., Fauvernier M., Fary C., Servien E., Lustig S., Batailler C. (2020). Total Hip Arthroplasty Performed by Direct Anterior Approach—Does Experience Influence the Learning Curve?. SICOT J..

[20] de Steiger R.N., Lorimer M., Solomon M. (2015). What Is the Learning Curve for the Anterior Approach for Total Hip Arthroplasty?. Clin. Orthop. Relat. Res..

[21] Di Martino A., Brunello M., Pederiva D., Schilardi F., Rossomando V., Cataldi P., D’Agostino C., Genco R., Faldini C. (2023). Fast Track Protocols and Early Rehabilitation after Surgery in Total Hip Arthroplasty: A Narrative Review. Clin. Pract..

[22] Rodriguez J.A., Deshmukh A.J., Rathod P.A., Greiz M.L., Deshmane P.P., Hepinstall M.S., Ranawat A.S. (2014). Does the Direct Anterior Approach in THA Offer Faster Rehabilitation and Comparable Safety to the Posterior Approach?. Clin. Orthop. Relat. Res..

[23] Klausmeier V., Lugade V., Jewett B.A., Collis D.K., Chou L.S. (2010). Is There Faster Recovery With an Anterior or Anterolateral THA? A Pilot Study. Clin. Orthop. Relat. Res..

[24] Barrett W.P., Turner S.E., Leopold J.P. (2013). Prospective Randomized Study of Direct Anterior vs Postero-Lateral Approach for Total Hip Arthroplasty. J. Arthroplast..

[25] Rathod P.A., Orishimo K.F., Kremenic I.J., Deshmukh A.J., Rodriguez J.A. (2014). Similar Improvement in Gait Parameters Following Direct Anterior & Posterior Approach Total Hip Arthroplasty. J. Arthroplast..

[26] Parvizi J., Restrepo C., Maltenfort M.G. (2016). Total Hip Arthroplasty Performed Through Direct Anterior Approach Provides Superior Early Outcome: Results of a Randomized, Prospective Study. Orthop. Clin. N. Am..

[27] Kucukdurmaz F., Sukeik M., Parvizi J. (2019). A Meta-Analysis Comparing the Direct Anterior with Other Approaches in Primary Total Hip Arthroplasty. Surgeon.

[28] Komnos G.A., Manrique J., Foltz C., Klement M.R., Restrepo C., Parvizi J. (2021). Transfusion Rates in Total Hip Arthroplasty Are Lower in Patients with Direct Anterior Approach. Arch. Bone Jt. Surg..

[29] Brunello M., Di Martino A., Ruta F., Ferri R., Rossomando V., D’Agostino C., Pederiva D., Schilardi F., Faldini C. (2023). Which Patient Benefit Most from Minimally Invasive Direct Anterior Approach Total Hip Arthroplasty in Terms of Perioperative Blood Loss? A Retrospective Comparative Study from a Cohort of Patients with Primary Degenerative Hips. Musculoskelet. Surg..

[30] Aggarwal V.K., Elbuluk A., Dundon J., Herrero C., Hernandez C., Vigdorchik J.M., Schwarzkopf R., Iorio R., Long W.J. (2019). Surgical Approach Significantly Affects the Complication Rates Associated with Total Hip Arthroplasty. Bone Jt. J..

[31] Goulding K., Beaulé P.E., Kim P.R., Fazekas A. (2010). Incidence of Lateral Femoral Cutaneous Nerve Neuropraxia after Anterior Approach Hip Arthroplasty. Clin. Orthop. Relat. Res..

[32] den Hartog Y.M., Mathijssen N.M.C., Peters S.J., Vehmeijer S.B.W. (2015). The Anterior Supine Intermuscular Approach for Total Hip Arthroplasty: Reducing the Complication Rate by Improving the Procedure. Hip Int..

[33] Lee G.C., Marconi D. (2015). Complications Following Direct Anterior Hip Procedures: Costs to Both Patients and Surgeons. J. Arthroplast..

[34] Haynes J.A., Hopper R.H., Ho H., McDonald J.F., Parks N.L., Hamilton W.G. (2022). Direct Anterior Approach for Primary Total Hip Arthroplasty Lowers the Risk of Dislocation Compared to the Posterior Approach: A Single Institution Experience. J. Arthroplast..

[35] Keggi K.J., Huo M.H., Zatorski L.E. (1993). Anterior Approach to Total Hip Replacement: Surgical Technique and Clinical Results of Our First One Thousand Cases Using Non-Cemented Prostheses. Yale J. Biol. Med..

[36] Nairn L., Gyemi L., Gouveia K., Ekhtiari S., Khanna V. (2021). The Learning Curve for the Direct Anterior Total Hip Arthroplasty: A Systematic Review. Int. Orthop..

[37] Di Martino A., Brunello M., Rossomando V., Pederiva D., Schilardi F., Stefanini N., Geraci G., Faldini C. (2023). Aesthetic Results, Functional Outcome and Radiographic Analysis in THA by Direct Anterior, Bikini and Postero-Lateral Approach: Is It Worth the Hassle?. J. Clin. Med..

[38] Lecoanet P., Vargas M., Pallaro J., Thelen T., Ribes C., Fabre T. (2018). Leg Length Discrepancy after Total Hip Arthroplasty: Can Leg Length Be Satisfactorily Controlled via Anterior Approach without a Traction Table? Evaluation in 56 Patients with EOS 3D. Orthop. Traumatol. Surg. Res..

[39] Sarraj M., Chen A., Ekhtiari S., Rubinger L. (2020). Traction Table versus Standard Table Total Hip Arthroplasty through the Direct Anterior Approach: A Systematic Review. HIP Int..

[40] Viamont-Guerra M.R., Chen A.F., Stirling P., Nover L., Guimarães R.P., Laude F. (2020). The Direct Anterior Approach for Total Hip Arthroplasty for Severe Dysplasia (Crowe III and IV) Provides Satisfactory Medium to Long-Term Outcomes. J. Arthroplast..

[41] Antoniadis A., Dimitriou D., Flury A., Wiedmer G., Hasler J., Helmy N. (2018). Is Direct Anterior Approach a Credible Option for Severely Obese Patients Undergoing Total Hip Arthroplasty? A Matched-Control, Retrospective, Clinical Study. J. Arthroplast..

